# Worth the Effort? Rehabilitation Causes and Outcomes and the Assessment of Post-Release Survival for Urban Wild Bird Admissions in a European Metropolis

**DOI:** 10.3390/ani15121746

**Published:** 2025-06-13

**Authors:** Marc Engler, Rebekka Sens, Maja Lundberg, Alexandra Delor, Marco Stelter, Malte Tschertner, Sina Feyer, Stephanie Zein, Lesley Halter-Gölkel, Rainer Altenkamp, Kerstin Müller

**Affiliations:** 1 NABU Landesverband Berlin e.V., Wollankstraße 4, 13187 Berlin, Germany; rsens@nabu-berlin.de (R.S.); mlundberg@nabu-berlin.de (M.L.); adelor@nabu-berlin.de (A.D.); mstelter@nabu-berlin.de (M.S.); raltenkamp@nabu-berlin.de (R.A.); 2 Independent Researcher, 10997 Berlin, Germany; m.tschertner@gmx.net; 3 Small Animal Clinic, School of Veterinary Medicine, Freie Universität Berlin, 14163 Berlin, Germany; sina.feyer@fu-berlin.de (S.F.); stephaniezein@gmx.de (S.Z.); lesleyhalter@web.de (L.H.-G.); kerstin.mueller@fu-berlin.de (K.M.)

**Keywords:** rehabilitation, post-release survival, wildlife rescue centre, admission cause, wild birds, conservation

## Abstract

Globally, millions of wild birds are admitted to rehabilitation centres each year. We analysed data on wild bird admissions at an urban rehabilitation centre in Berlin, Germany, collected over 20 years (2005–2024), aiming to (a) characterise admission causes and demographics, (b) investigate the rehabilitation duration and release probability across admission causes and systematic bird groups, and (c) assess post-release survival as a proxy for rehabilitation success. Longer rehabilitation durations were generally associated with orphaned birds and those in poor condition or who had had an infection. Orphans and birds with undetermined admission causes were most likely to be released. Birds that were admitted in poor condition were least likely to be released, which was particularly the case among Passerines. The monitoring of post-release survival through ring recovery data revealed higher recovery rates for larger birds but no informative value on post-release survival across species; thus, it did not represent an ideal measure of rehabilitation success in terms of the original objective. We conclude that the extent, outcome, and success of wild bird rehabilitation may depend on the initial cause of admission and may differ between bird groups. Advanced measures to assess post-release survival should be considered to allocate the limited resources and the conservation efforts of wild bird rehabilitation centres to birds of species and/or admission causes best suited to undergoing rehabilitation.

## Simple Summary

Globally, millions of wild birds are admitted to rehabilitation centres each year. We analysed data on wild birds admitted to an urban rehabilitation centre in Berlin, Germany, collected over 20 years (2005–2024), aiming to (a) investigate why different bird groups were admitted, (b) understand how the bird group or the reason for admission influenced the rehabilitation duration and whether a bird could be released again, and (c) assess survival after release as an indication for rehabilitation success. Birds spent more time in rehabilitation if they were orphans or admitted in poor condition or with an infection. Orphans and birds with an unknown background were most likely to be released. Birds that were admitted in poor condition were least likely to be released; this was particularly the case among songbirds. How long birds stay in rehabilitation and whether they can be released again may depend on the reason why they are admitted and may differ between systematic bird groups. Based on re-sightings of released birds with individual rings around their legs, we found that larger birds were re-sighted more often than small or medium-sized birds. However, we received so little information regarding whether birds survived after release that we could not draw any conclusions on rehabilitation success using this method. Better measures to observe whether birds survive after release should be considered so that the limited resources and time available to wild bird rehabilitation centres can be invested in the species and/or admission causes with the best outcomes.

## 1. Introduction

Globally, wildlife rehabilitation is responsible for the rescue, temporary care, and release of millions of animals each year [1]. Wildlife rehabilitation centres (WRCs) are often welfare-driven, but they can also provide a public educational service by enhancing the awareness and understanding of anthropogenic threats to wildlife among citizens [2,3,4]. Furthermore, WRCs may contribute to the monitoring and evaluation of population status and novel threats by providing long-term, on-the-ground records for admitted individuals [5,6,7]. Moreover, wildlife rehabilitation has been hypothesised to reinforce existing populations after release, particularly in species that are of conservation concern [1,8,9,10].

In urban environments, increased levels of soil sealing, elevated traffic volumes, and a greater density of human-built structures compared to natural habitats pose significant threats to wildlife. For example, birds face higher risks of colliding with vehicles, trains, or buildings in urban areas [11,12,13,14]. In Germany alone, an estimated 15 million birds perish due to collisions with glass every year [15]. In addition, attacks by free-ranging domestic cats have been shown to account for a significant share of admissions to WRCs in the UK and North America, particularly during the breeding season [16,17,18]. Furthermore, the risk of exposure to potentially harmful environmental pollutants [19,20] or diseases [21,22] can be elevated in urban environments.

Under these regional conditions, the systematic release of rehabilitated individuals has the potential to serve as a mitigating mechanism against the deleterious effects of human activity on wildlife, particularly among bird species such as raptors, which often display a slow life cycle and low population densities [8,14,23,24]. Among other methods, WRC admission data can thereby significantly contribute to quantifying and understanding the effects of anthropogenic threats on urban birds [14,25].

Complete and consistent long-term datasets on wildlife admissions, the rehabilitation process, and their outcomes are utilised by wildlife rehabilitators to plan and provide appropriate resources and to draw conclusions to inform future strategies, especially when funding is limited [18]. The assessment of such data enables WRCs to focus their efforts and resources on measures that offer the greatest benefit–cost ratios and/or those that increase the likelihood of release [26].

It is essential that wildlife rehabilitation programmes implement transparent measures to evaluate their effectiveness or success. To begin with, these evaluations depend on the definition of these terms [26]. For instance, the term “success” could constitute any definition, from recovery from an injury and subsequent release on the individual level to the broader maintenance of population stability through the release of rehabilitated animals [27]. The definition of success can further depend on the temporal scale of post-release survival (e.g., short-term vs. long-term survival). The methods employed to verify and quantify post-release survival, such as individual marking with rings or the deployment of GPS transmitters, can yield data of inconsistent quality, necessitating appropriate interpretation [27,28,29].

In this study, we compiled and analysed wild bird admission data for an urban WRC in the largest German metropolis, collected over a 20-year period (2005–2024). The objective of this study was threefold: (1) to characterise the causes and species-level demographics of admissions; (2) to investigate whether rehabilitation duration and the probability of release (i.e., rehabilitation outcome) depend on the interplay between the cause of admission and systematic bird group; and (3) to evaluate the post-release survival of rehabilitated individuals as a proxy for rehabilitation success, with the goal of drawing conclusions to inform future rehabilitation strategies.

## 2. Material and Methods

### 2.1. Study Background and Area

**Figure 1:**
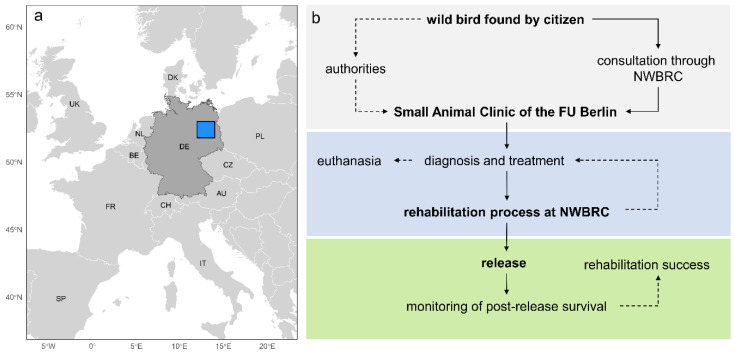
Location of the study area and schematic representation of the rehabilitation process for wild birds admitted to the Wild Bird Rehabilitation Center of the NABU Berlin. (**a**) The study area (blue box) comprises the city area of Berlin in North-East Germany. (**b**) Schematic representation of the rehabilitation process, from the moment that a wild bird is found within the city area to the monitoring of the individual after release. Euthanasia, humanely inducing death in an animal with minimal pain and distress; FU Berlin, Freie Universität of Berlin; NWBRC, Wild Bird Rehabilitation Center of the NABU Berlin.

The Wild Bird Rehabilitation Center (hereafter NWBRC) of the NABU Berlin e.V. (Nature And Biodiversity Conservation Union) is located in the eastern part of the German capital, Berlin (Germany, N52.50343–N53.53618, E13.40114–E13.41122, Figure 1a). Berlin has an estimated population size of 3.87 million people within an area of 891 km^2^. The city landscape is characterised by significant heterogeneity, with highly urbanised areas and approximately 60% of the city surface sealed and dedicated to housing and traffic. The city’s extensive green infrastructure, comprising 2500 city parks and 160 square kilometres of forest, contributes to the overall green space within the urban area. Water bodies and streams account for ca. 6% (54 km^2^) of the city’s total area (“www.statistik-berlin-brandenburg.de (accessed on 11 January 2024)”).

Since 1998, the NWBRC has been running a year-round rehabilitation programme for wild bird species. This programme includes consultations with citizens and veterinary diagnosis and treatment for birds in need, as well as their rehabilitation until release (Figure 1b). An extensive telephone consultation service is provided via an emergency hotline for concerned citizens and authorities in cases of found birds and for all other wild bird-related topics. Wild birds found within the study area (ca. 2 km buffer around the administrative boundaries of Berlin) and in need of veterinary support are transferred to the Small Animal Clinic of the Freie Universität Berlin, which serves as the main point of contact for injured wildlife in Berlin (Figure 1b). The veterinary care of the birds includes a thorough clinical examination, with further diagnostics implemented on a case-by-case basis, e.g., radiography, ultrasonography, computed tomography, blood and faecal examination, the cytological examination of crop smears and punctuates, and microbiological examinations. The initiation of treatment is contingent upon the species, age, and clinical findings. In cases of severe injury or illness with no realistic chance of successful rehabilitation, the animal is euthanised.

Once veterinary treatment is completed, birds with a realistic chance of successful release are transferred to the NWBRC facilities for further rehabilitation. Animals are then cared for by the staff at NWBRC. Factors such as autonomous feed intake, stable species-specific body mass, flight capability, and the presence of species-specific behaviours are considered to determine the time of release.

### 2.2. Data Collection

The admission records of NWBRC were collected over a period of 20 years, between 2005 and 2024, in a Microsoft Access database. For each individual, parameters including the (a) species, (b) age (juvenile/adult), (c) time and location of the incident (e.g., the calendar year, admission date, coordinates, and type of location), (d) cause of admission, and (e) outcome (released or deceased/euthanised, time and location of release) were collected by trained staff. The objective of this study was to examine the interplay among the admission cause, bird group, and duration/outcome of rehabilitation. Consequently, the dataset for admissions exclusively included individuals who, from a clinical perspective, had a realistic chance of successful rehabilitation in the first place. Thus, we excluded individuals who were admitted to the clinic but had to be euthanised due to a low possibility of successful rehabilitation. Furthermore, we excluded all animals that were handled through in-field services rather than admitted to the clinic, as these do not reflect the typical rehabilitation process (Figure 1b). Examples of this include the relocation of Mallard Duck (*Anas platyrhynchos*) families that had been nesting on anthropogenic structures [5] and adoption programmes for Common Swifts (*Apus apus*).

### 2.3. Causes and Demographics of Admissions

We classified the cause of admissions based on the diagnosis and admission notes collected for each individual to represent a combination of clinical symptoms at admission and the underlying cause for which an individual was initially admitted. To increase comparability across studies, we followed the classification process of similar, recent studies as closely as possible [14,25,30] and grouped admissions into eight different “causes”: (a) “anthropogenic structure”, (b) “building collision”, (c) “vehicle collision”, (d) “unknown trauma”, (e) “persecution”, (f) “pet attack”, (g) “poor overall condition”, (h) “infection”, (i) “orphaned”, and (j) “undetermined” (see Table S1 for detailed definitions of admission causes). The admission cause “undetermined” included all cases of individuals without diagnosed injuries and where no particular cause could be determined.

Although some admission causes evidently reflect direct human impact (e.g., vehicle or building collisions), we opted against grouping admission causes into more general types of admission, e.g., either “anthropogenic” or “natural”, as implemented in some previous studies [14]. This approach was adopted because we recognise that for certain admission causes, a high degree of uncertainty remains as regards the true course that led to the need for veterinary support in an individual. Consequently, any conclusions drawn about anthropogenic impacts may be affected by reasonable bias. For instance, “poor overall condition” was considered as the cause of admission if individuals were admitted with signs of dehydration, starvation, and/or apathy, but whether this was naturally or anthropogenically induced remains unknown.

Furthermore, we grouped wild bird admissions into six bird groups to reflect practical admission scenarios and common ecological niches rather than strict systematic bird orders. In addition to the groups of Accipitriformes, Falconiformes, and Strigiformes, we distinguished between “Corvids” (i.e., the Carrion Crow (*Corvus corone*)*,* Eurasian Jay (*Garrulus glandarius*), Eurasian Magpie (*Pica pica*) and Western Jackdaw (*Corvus monedula*)) and all other “Passerines” and designated the species “Wood Pigeon” (*Columba palumbus*) as a separate group due to its substantial sample size. With the exception of a single species (Redwing (*Turdus iliacus*); status, “Near-Threatened”), all species included in this paper are classified as having an extinction risk status of “Least Concern” [31]. The data collection for admissions did not include any bird species classified as “invasive” during the time period investigated.

### 2.4. Rehabilitation Duration and Outcome for Admissions

In order to investigate variability in the duration of rehabilitation from admission to release, we fitted a generalised linear model (GLM) predicting rehabilitation duration as a function of admission cause and systematic bird group. For admissions with the outcome “deceased” or “euthanised”, the sample sizes for admission cause–bird group level combinations were too low to comprehensively model the rehabilitation duration for these two outcomes. We thus excluded admissions that died (*n* = 764) or were euthanised (*n* = 722) during the rehabilitation process from the analysis, and we instead report descriptive metrics for the rehabilitation duration for these two groups. For birds with the outcome “released”, we calculated “rehabilitation duration” as the number of days between the date of admission to the clinic and the date of release to ensure a comprehensive assessment of the entire rehabilitation process. The distribution for rehabilitation duration was further right-skewed due to a few individuals with very long rehabilitation durations of over 200 days (see Figure S1). This was largely attributable to birds with severe plumage damage or releases that were postponed due to extreme weather periods (*n* = 40). To reduce bias, these individuals were excluded from the analysis, and the median absolute deviation (MAD) was used to report central measures of the variable rehabilitation duration. To avoid separation among combinations of linear predictors, we pooled the three bird orders of “Accipitriformes”, “Falconiformes”, and “Strigiformes” in a new group, termed “Raptors”, considering that they represent similar ecological niches. Due to the low sample sizes for some bird group–admission cause combinations, we further removed the admission causes “persecution” and “pet attack” from our analysis. The final dataset used to predict rehabilitation duration thus included a total of 3510 admissions.

We fitted a zero-truncated model with a negative binomial error distribution and “logit” link function to account for overdispersion resulting from the absence of zero values in the response variable (e.g., a minimum rehabilitation duration of one day). We implemented an interaction effect between admission cause and bird group in the model structure and chose “undetermined” and “Passerines” as the respective reference levels for admission causes and bird groups. To quantify the work capacities invested in each species over the entire study period, we also reported the species-specific “rehabilitation effort” as the cumulative rehabilitation duration (days) for all individuals of a given species.

Furthermore, we investigated whether the probability of a bird being released was related to the initial cause of admission or its systematic bird group. We grouped the outcomes of admissions into two categories by creating a binary variable “outcome” (0 = bird deceased/euthanised, termed “deceased” and 1 = bird released, termed “released”; see [14]). We then modelled the release probability as the response variable using the admission cause and bird group, as well as their interaction, as explanatory variables in a GLM with logistic regression with binomial error distribution and “logit” link function. The model was fitted on a dataset that excluded the admission causes “persecution” and “pet attack” (see above) and pooled all raptor species in a new group, “Raptors”. The final dataset used to predict release probability thus included a total of 4981 admissions. The reference levels for admission cause and bird group were designated as “undetermined” and “Passerines”, respectively.

### 2.5. Assessment of Post-Release Survival and Rehabilitation Success

In order to facilitate the post-release monitoring of rehabilitated birds and evaluate their survival as a validation measure for rehabilitation success, a subset of individuals were ringed with individually coded, non-coloured metal rings (sizes F-K and inner diameter of 5.5–13 mm based on species) provided by the ringing scheme Radolfzell (Germany) around the tarsometatarsus prior to release [32]. The marking of birds allowed for the identification of individuals post-release, including the reading of the ring in cases of recapture or distant reading, as well as when a freshly deceased bird (e.g., death within 5 days) was found. We exported reports of ring recovery at the end of 2024 (31 December 2024) from the ringing scheme Radolfzell for all individuals that were marked between the 1 January 2005 and the 30 June 2024. The latter date was chosen to ensure that ring recovery events for released birds were feasible for a minimum period of six months after release.

First, we calculated ring recovery rates as the proportion of individuals recovered out of the total number of ringed individuals for each species and systematic bird group. To assess rehabilitation success at the species level, we exclusively included species with a minimum of 10 ringed and released individuals throughout the entire study period. Conclusively, from the full data set comprising 1789 ringed individuals from 24 species, we excluded 22 individuals from 12 species. For the resulting data set, we considered the rehabilitation of an individual successful if it was verified as having survived for a minimum of two months after release (61 days = “post-release short-term survival”, [26], which was confirmed through its state (dead/alive) at, and the timing (>/<61 days) of, recovery. Individuals that were recovered alive prior to the specified threshold or had no recorded post-release recovery events were classified as “unknown” [26].

### 2.6. Statistical Analysis

All GLMs were fitted using the function *fitme* in the R package *spaMM* version 4.5.0, with the method set to “PQL/L” [33]. We tested the significance of variables for the selected model using parametric bootstrap on 1000 replicates, as implemented by the function *LRT* from *spaMM*. We tested all main model assumptions, including linearity, uniformity, serial autocorrelation, and overdispersion, by simulating residuals via parametric bootstrapping using the R package *DHARMa* version 0.4.7 [34]. Data processing and statistical analysis were performed in R version 4.4.3 [35].

## 3. Results

### 3.1. Causes and Demographics of Admissions

**Figure 2:**
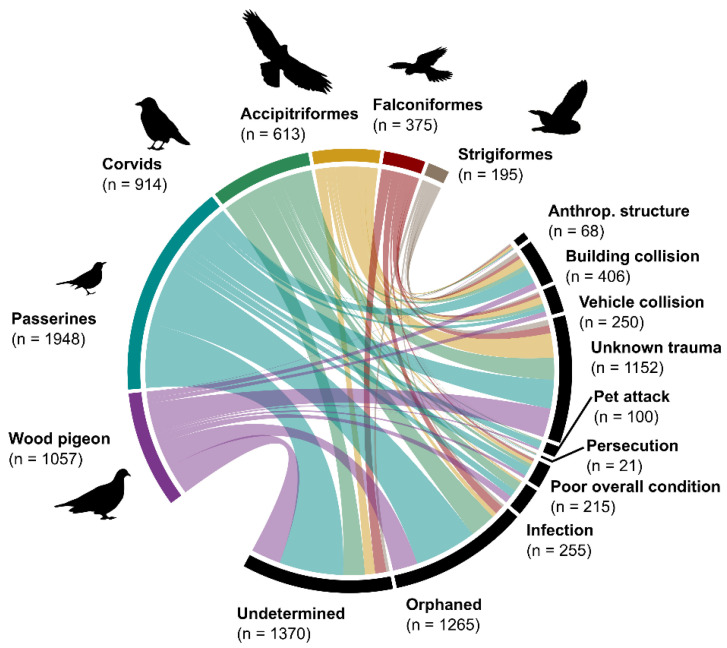
Admission causes for five groups of wild birds admitted to NWBRC between 2005 and 2024 (*n* = 5102). Admissions were grouped based on systematic bird groups (top left panels), and admission causes (bottom right panels), with the field size proportional to the share of all admissions per order or admission cause, respectively.

Across the 20-year study period, a total of 5102 wild birds of 78 species were admitted to NWBRC. The majority of these admissions were Passerine species (38.2%, *n* = 1948, *n*_species_ = 50), the Wood Pigeon (20.7%, *n* = 1057), and Corvids (17.9% of admissions, *n* = 914, *n*_species_ = 5), which accounted for more than 75% of admissions (Figure 2). Across species, the Wood Pigeon was the most numerous (20.7%, *n* = 1057), followed by the Carrion Crow (11.1%, *n* = 565) and the House Sparrow (*Passer domesticus*, 10.5%, *n* = 537, Table 1).

The most prevalent causes of admissions were “undetermined” (*n* = 1370, 26.9%) and “orphaned” (*n* = 1265, 24.8%), accounting for more than half of all admissions (Table 1). Admissions associated with collisions with anthropogenic structures (*n* = 68, 1.3%), buildings (*n* = 406, 8.0%), or vehicles (*n* = 250, 4.9%) or involving “unknown trauma” (*n* = 1152, 22.6%) accounted for more than one-third of admissions. Other causes accounted for 11.5% of admissions (*n* = 591; see Table 1).

At the species level, Wood Pigeons accounted for the majority of orphaned admissions (*n* = 243, 19.2%), followed by the House Sparrow (*n* = 186, 14.7%) and Carrion Crow (*n* = 166, 13.1%). Among raptor species (Accipitriformes, Falconiformes, and Strigiformes), the majority of individuals were admitted due to unknown trauma (*n* = 370, 31.3%) or collisions with buildings (*n* = 153, 12.9%), vehicles (*n* = 121, 10.2%), or anthropogenic structures (*n* = 51, 4.3%; see Figure 2).

Throughout the study period, the rehabilitation efforts of NWBRC totalled 158.741 days, as measured by the cumulative rehabilitation duration of all individuals. Of these, Passerines accounted for more than a third of the overall rehabilitation effort (33%, *n* = 52.117 days, Table 1). Raptor species constituted 13.1% (*n* = 23.979 days) of the cumulative rehabilitation effort (Table 1). On the species level, Wood Pigeons alone accounted for almost one-third of the overall rehabilitation effort throughout the study period (32%, *n* = 50.065 days).

### 3.2. Rehabilitation Duration for Admissions

Across species and admission causes, birds that were deceased during the rehabilitation process had a median rehabilitation duration of 11 days (±12, range = 1–202, *n* = 764). Likewise, birds were euthanised by the clinic veterinarians after a median rehabilitation duration of 8 days (±10, range = 1–171, *n* = 764) in cases with no realistic prognosis for successful rehabilitation.

The median duration for admitted individuals to reach complete rehabilitation with the outcome “released” was 36 days (±22, *n* = 3616), with a range from 1 to 197 days (Table 2). To understand the factors influencing the duration of rehabilitation, we ran a GLM to predict the rehabilitation duration based on the interaction between the bird group and the admission cause. The model was statistically significant (LRT_PQL/L_ = 1119.2, df = 31, *p* < 0.001), and we found that the duration of rehabilitation was influenced by the interaction between the admission cause and bird group, thus predicting the rehabilitation duration significantly better than a model containing both predictors as single fixed effects without their interaction (LRT_PQL/L_ = 202.2, df = 21, *p* < 0.001; see Table S2 for estimate details).

**Figure 3:**
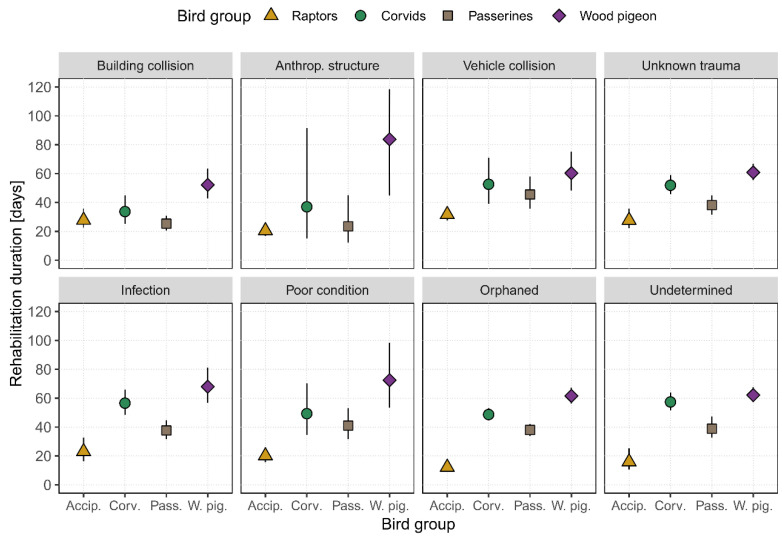
Predicted rehabilitation duration [days] for eight causes of wild birds being admitted to NWBRC between 2005 and 2024 (*n* = 3510). The underlying data set only included individuals who were released and thus represented the full rehabilitation process. Symbols represent estimates coloured and shaped according to the bird group, with error bars representing 95% confidence intervals.

Among bird groups, the rehabilitation duration was shortest for raptors for all admission causes except those resulting from building collisions. Conversely, the duration of rehabilitation was found to be the longest for Wood Pigeons across all admission causes (Figure 3). For Wood Pigeons, the rehabilitation duration was particularly prolonged when they were admitted as orphaned (61.5 days, CI_95%_ = 56.2–67.4), in poor overall condition (72.5 days, CI_95%_ = 53.3–98.5), or following infections (68.0 days, CI_95%_ = 56.8–81.3; see Table S2 for model estimates). Birds admitted due to anthropogenic structures showed the highest variability in rehabilitation duration, particularly in the case of Corvids (37.0 days, CI_95%_ = 15.0–91.7) and Wood Pigeons (83.8 days, CI_95%_ = 44.6–119.7). For Passerines, the rehabilitation duration ranged from 23.5 to 45.5 days (CI_95%_ = 12.3–57.9), depending on the underlying cause of admission.

### 3.3. Outcomes of Rehabilitation

Of all admissions considered in this study, a total of 71% (*n* = 3616) resulted in the release of birds after rehabilitation, while 29% (*n* = 1486) died or had to be euthanised during the rehabilitation process (Table 2). To further investigate the influence of the bird group and the cause of admission on the likelihood of release, we ran a GLM to predict release probability based on the interaction between the two predictors. We found that the model was statistically significant (LRT_PQL/L_ = 263.4, df = 31, *p* < 0.001) and that the probability of release was further influenced by the interaction between the admission cause and bird group (LRT_PQL/L_ = 33.3, df = 21, *p* < 0.04; see Table S3 for estimate details). Thus, the model predicted release probability significantly better than a model including both predictors as independent fixed effects.

**Figure 4:**
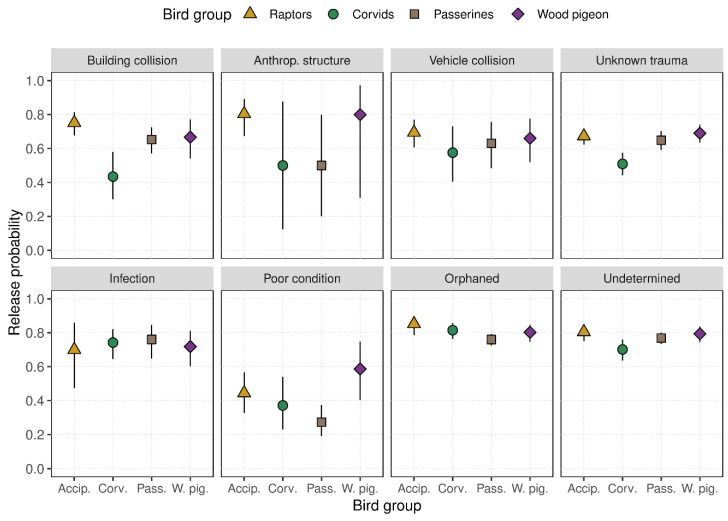
Predicted release probability for eight causes of wild birds being admitted to NWBRC for rehabilitation between 2005 and 2024 (*n* = 4981). Symbols represent estimates coloured and shaped according to the bird group, with error bars representing 95% confidence intervals.

Compared to all other admission causes and consistently across bird groups, admissions of either orphaned individuals or those where the cause could not be determined yielded the highest estimates for release probability, exceeding 70% across all group–cause combinations. In contrast, when considering admissions due to collision with anthropogenic structures, we found the highest variability in predicted release probabilities, with estimates ranging between 0.5 and 0.8 (CI_95%_ range = 0.12–0.97). In cases where birds were admitted after collisions with buildings, release probabilities were particularly high for raptor species (0.75, CI_95%_ = 0.68–0.81) but especially low in the group Corvids (0.43, CI_95%_ = 0.30–0.58). Birds that were initially admitted in poor overall condition were the least likely to be released, particularly among the group Passerines (0.27, CI_95%_ = 0.19–0.37). In contrast, Wood Pigeons demonstrated the highest probability estimates for release in this admission category (0.59, CI_95%_ = 0.40–0.75) (Figure 4).

### 3.4. Post-Release Survival and Rehabilitation Success

**Figure 5:**
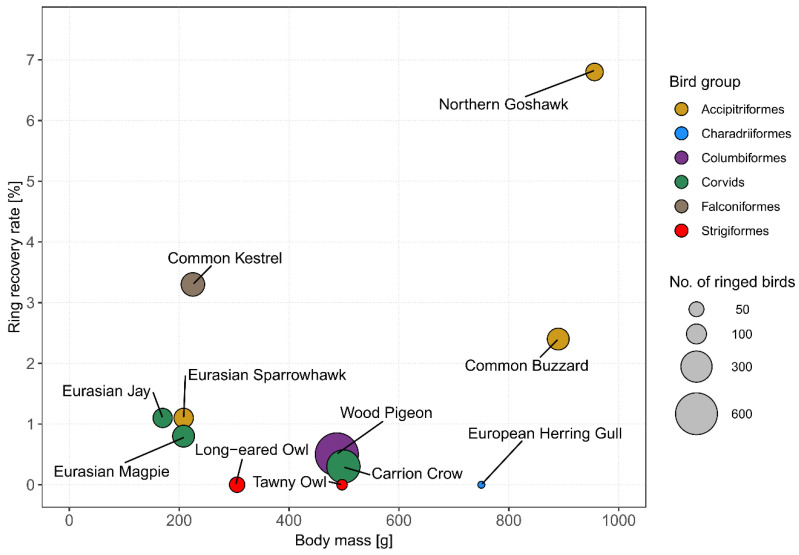
Relationship between ring recovery rate (percentage of recovered individuals after release) and species-specific body mass. Individual circles represent different bird species, with the circle area proportional to the number of ringed birds per species (range = 15–641 individuals). For better display and scale, the species Grey Heron is not shown in the plot (*n* = 15, body mass = 1500 g, ring recovery rate = 26.7%).

We analysed ring recovery data for a total of 1767 ringed and released birds of 12 species to assess post-release survival as a proxy for rehabilitation success. Throughout the study period, a total of 24 ring recoveries involving 24 individuals across nine species were documented (Table 3). The mean ring recovery rate across species was 1.3%, and species-specific ring recovery rates were generally associated with a higher average body mass (Figure 5). The highest ring recovery rates were observed in the Grey Heron (*Ardea cinerea*) at 26.7%, followed by frequently admitted raptor species such as the Northern Goshawk (*Accipiter gentilis*, 6.8%) and the Common Kestrel (*Falco tinnunculus*, 3.3%). Despite showing the highest number of ringed and released individuals, Wood Pigeons yielded one of the lowest ring recovery rates (0.5%), with only 3 out of 641 individuals being recovered after release.

Across all species, post-release survival could be verified in only 1% (*n* = 19) of individuals, while for 99% of ringed rehabilitated birds (*n* = 1748), it remained unknown whether they had survived for a minimum of two months after release (Table 3). Consequently, the conclusions that could be drawn about the success of rehabilitating released birds were very limited. In 16 out of the 1767 cases, rehabilitation was verified as being successful. In three cases, rehabilitation was verified as not being successful. For Wood Pigeons, the rehabilitation success could only be verified in 1 out of 641 cases, which turned out to have failed. For four species, the assessment of rehabilitation success was precluded due to the lack of verified post-release survival events; i.e., the status of post-release survival remained unknown in all cases (Figure 5, Table 3).

## 4. Discussion

In this study, we investigated the demographics, causes, and outcomes of bird admissions in different bird groups to a European urban rehabilitation centre and analysed factors influencing the rehabilitation process and outcome. We discuss, in turn, the results regarding the causes and demographics of admissions, the rehabilitation duration and outcome, and measures to assess post-release survival before concluding by considering their implications for future rehabilitation strategies.

This study was based on the admission data of birds that were assessed as having a realistic chance of successful release and were, therefore, pre-selected, so it did not include all admissions to the veterinary clinic. This was because the aim of this study was to look specifically at the rehabilitation process of birds admitted due to different causes and draw conclusions about which cases were likely to be successfully rehabilitated rather than investigating the clinical condition and rate of euthanasia/survival at the first examination. We thus emphasise that the results and comparisons with other studies should be interpreted accordingly [26].

### 4.1. Causes and Demographics of Admissions

In general, causes of admissions can vary considerably between geographical regions and different habitat types, for instance, along the urban–rural gradient [36,37] or between landbound and coastal regions [24,27]. Given that our study area represented a highly urban habitat with a strong overall human presence, we can also generally assume that there are increased chances of birds being found by citizens and thus admitted to veterinary institutions. However, like many recent studies conducted in the UK [14], Spain [25], and Greece [38], the underlying cause of admission remains unknown for almost one-third of admissions in this study. This includes cases with undetermined causes or those admitted with unknown trauma or in poor overall condition. Consequently, it is likely that admissions initially caused by human influence are generally underestimated in terms of absolute numbers, particularly due to the lack of available information for the staff at NWBRC. Furthermore, birds suffering from severe trauma through persecution, collisions, or intoxication may perish on site or shortly afterwards and/or are often not reported, therefore introducing additional bias among the causes of admissions [15].

Admissions following unknown trauma or that were caused by collisions with buildings, vehicles, or anthropogenic structures accounted for more than one-third of wild bird admissions. This is in line with numerous other studies that have identified these causes as serious challenges for wild birds, particularly in larger species such as raptors [24,39] and especially in urban areas [14,40]. Projections indicate a steady rise in human population beyond 2035, not only in Berlin but also in the majority of European metropolises. This is expected to result in an increase in urbanisation, driven by the demand for residential areas and infrastructure. Concurrently, increasing levels of traffic volume, soil sealing, and building density may further increase the probability of collision with artificial structures, thereby amplifying these challenges in the future [41].

We found that almost a quarter of birds were admitted as “orphaned”, which is similar to results reported in previous studies covering multiple bird families [42,43] but lower than those from studies targeting specific species groups, e.g., kestrels [44] and waterfowl [40]. We emphasise that for the majority of “orphaned” admissions, the underlying cause that led to this situation remains unknown [18]. It is likely that the majority of these admissions comprised nestlings that fell out of the nest prematurely (due to, for example, poorly constructed nests or strong winds) and were thus found by citizens. For many WRCs, the lack of information about the circumstances at the location of the find, coupled with there commonly being a delay in reporting the find to the WRC, makes an immediate return to the site of origin impossible, leaving hand rearing as the only option [45].

In this study, Wood Pigeons accounted for the most “orphaned” individuals and represented the largest number of individuals across all species. Often admitted at the nestling age, the rehabilitation process to reach independence is long, and it, therefore, yielded the highest rehabilitation effort across species, accounting for almost a third of the overall workload of the NWBRC throughout the study period. This is not surprising as Wood Pigeons are abundant in many European cities, often nesting in residential areas near humans and are particularly susceptible to premature nest failure, e.g., due to the highly abundant Carrion Crow or poorly constructed nests [45,46].

### 4.2. Rehabilitation Duration and Outcomes of Admissions

In our study, more than two-thirds of rehabilitated individuals were released, regardless of the reason for admission or the bird group. Many comparable studies in Europe [8,14,18], the USA [39,40], and Australia [43] have reported lower release and higher mortality rates. However, differences in intervention-related factors and the pre-selection of data should be taken into account when comparing study results [26]. Depending on the stages of admission/rehabilitation included in the admission data analysed, the reported release probabilities may be lower (i.e., if cases of euthanasia on admission are included, e.g., [47]) or even higher than in our study (i.e., if birds are transferred to other facilities and considered as rehabilitated per se, e.g., [37]).

Our analysis revealed that the duration of the rehabilitation process and the likelihood of being released were both shaped by the interplay between the cause of admission and the bird group. The statistical significance of the interaction term highlights that the effect of the admission cause on the outcome and duration was not uniform across bird groups (and vice versa). We found that orphaned birds were the most likely of all bird groups to be released, which is consistent with many previous studies [14,38,40,44]. Many of these birds were probably not true orphans but were easily found by people in the urban area and, in most cases, were released due to their good overall initial health condition [47]. Although background information on the circumstances and location of origin can generally contribute to a faster diagnosis and a more appropriate rehabilitation plan, birds admitted with an undetermined cause showed almost identical patterns to orphaned birds in terms of rehabilitation duration and release probability. We suggest that bias in the initial grouping due to a lack of information may be the cause. Thus, a considerable proportion of birds with an undetermined cause of admittance were probably admitted as orphaned/juveniles but could not be identified as such.

We observed the greatest variation in both rehabilitation duration and release probability for birds in contact with anthropogenic structures. We conclude that this can be attributed to a wide range of clinical patterns, ranging from minor soft tissue injuries (e.g., after entanglement) to long-term plumage damage (e.g., after collisions with fences). Across all bird groups, individuals in poor overall condition at the start of the rehabilitation process were least likely to be released, which is consistent with other studies (e.g., [14]). While a variety of individual-related factors have been identified across studies as influencing survival during rehabilitation, including age, body mass, and species-specific traits [26], poor rescue conditions alone can reduce the likelihood of release [45,48]. However, the clinical diagnosis and the initial condition of the admitted bird are often multifactorial and can vary widely, even within admission groups [49]. In fact, the true underlying cause for birds in this category is multifactorial and likely pools individuals from all other categories of admission causes. Nevertheless, this group represents one of the most common scenarios faced by wildlife rehabilitators in daily practice and appears to be only marginally profitable, at least for the bird groups Corvids and Passerines.

Persecuted birds, especially raptors, often suffer from more severe symptoms due to gunshots or trapping incidents [24,47]. However, such incidents were very rare in our study, presumably due to the highly urban environment and low persecution levels in our study region, and we were, therefore, unable to examine the rehabilitation duration and release probability for this admission group [50,51].

### 4.3. Post-Release Survival and Rehabilitation Success

In order to verify post-release survival as a measure of rehabilitation success, we examined the ring recovery data of rehabilitated and released birds. We found that higher ring recovery rates were generally associated with a higher body mass, which is presumably due to the increased probability of ring recovery and consistent with similar studies in the USA [52] and the UK [53,54]. Species-specific ring recovery rates in this study were still lower than most other studies using metal rings to individually mark released birds, including various raptor species [52,55], Cape vultures (*Gyps coprotheres*) [9], and California Brown Pelicans (*Pelecanus occidentalis californicus*) [56]. This was probably due to the fact that all of these studies either used additional coloured rings with an increased recovery probability or were based on significantly larger sample sizes, i.e., on a national scale. Although this study was conducted in a highly urban environment with a very high human population density, the ring recovery rates were too low to adequately assess post-release survival as a measure of rehabilitation success. We consider it unlikely that rapid scavenging [57] or the reluctance of citizens to report ring recoveries to the ringing scheme particularly contributed to lower recovery rates compared to the generally low rates in both urban and rural areas [54]. For the vast majority of released individuals, it remains unknown whether the rehabilitation process was successful in terms of short-term survival [26]. However, it is possible that a reasonable proportion of rehabilitated birds were predated shortly after release, at least in the Passerine group, Wood Pigeons, and some Corvid species. In the specific case of “orphaned birds”, it additionally remains unknown to what extent the lack of natural parental care during rearing in WRCs may affect short- or long-term survival after release.

In particular, in the case of the Wood Pigeon, the ringing of released birds did not allow an adequate assessment of rehabilitation success, as post-release survival could only be verified in 1 out of 641 cases. We suspect that this could have been caused by several factors, including their high vigilance and thus distance from humans and their small rings in combination with relatively short tarsi. Potentially, people were also more likely to ignore dead Wood Pigeons compared to more unusual species because they are more common and an expected sight in urban areas. Nevertheless, Wood Pigeons were the most common species admitted to NWBRC during the study period and had one of the highest median rehabilitation durations, accounting for almost a third of the NWBRC’s total workload. The effort invested in rehabilitating this care-intensive species is considerable, but the question remains as to what percentage of these birds survive after release and whether the effort invested is producing the desired outcomes. Comparable studies addressing this dilemma are virtually non-existent. Only in the UK has it been reported that rehabilitated Wood Pigeons largely survive for more than eight weeks, but the results should be interpreted with caution, as they were also based on only a few ring recoveries (*n* = 15, ring recovery rate = 1.4% [42]).

Given the small number of recovery events and verified cases of rehabilitation success in this study, we report and discuss the post-release survival of rehabilitated birds only at the species level. Unfortunately, we were not able to analyse and interpret differences between causes of admission, let alone clinical patterns and conditions, that may have influenced the likelihood of post-release survival. However, reviewing the outcome of the medical treatment regimens applied provides the basis for (a) continuously improving the quality of the rehabilitation process, (b) assessing whether the initiation of treatment is appropriate from the perspective of animal welfare, and (c) further improving the allocation of resources to the most promising cases.

Recent studies have used more advanced methods such as GPS transmitters to assess the post-release survival of rehabilitated birds, including the White-Tailed Eagle (*Haliaeetus albicilla*) [58], Red-Tailed Hawk (*Buteo jamaicensis*) [59] and Crested Ibis (*Nipponia nippon*) [29]. Although costly, GPS transmitters provide highly reliable data, allowing rehabilitation success to be verified beyond post-release survival. The ability to track the post-release life course of rehabilitated birds, in terms of changes in social behaviour, spatial use, and even reproduction, compared to non-rehabilitated birds, may facilitate the assessment of rehabilitation success at scales that are currently largely unexplored [26,27].

## 5. Conclusions

This study used long-term data on wild bird admissions from a highly urban environment to analyse the causes and outcomes of rehabilitation and to evaluate rehabilitation success through post-release monitoring. This study was motivated by the fact that every year, the NWBRC devotes significant amounts of their resources to the rehabilitation of specific groups of wild birds. If this use of resources were proven to be unprofitable in terms of post-release survival, then the resources could be invested in other individuals or species with a better chance of successful rehabilitation.

Our long-term data show that urban wild bird admissions are often associated with some degree of human impact and that rehabilitation duration and the likelihood of release vary widely among admission causes and bird groups. As human impacts on wildlife will probably increase in the future, rehabilitation data represent a valuable resource for identifying trends in threats to all wildlife. For WRCs, post-release monitoring is essential in optimising treatment regimens and assessing whether resources are being directed towards useful actions and contributing to the original objective. While the ringing of rehabilitated birds is a common practice in WRCs for post-release monitoring, at least in the context of our study, the resulting information on post-release survival is very limited, suggesting that ringing does not represent an ideal method for assessing rehabilitation success. With limited resources in terms of funding, staff, and time in almost all WRCs worldwide, WRC staff should consider methods that are both reliable and realistic to prioritise conservation efforts for the species/groups best suited for rehabilitation in terms of animal welfare and post-release survival. This is particularly important if WRCs are investing a large proportion of their resources in care-intensive birds of a single species, thereby compromising their efforts in other conservation tasks. Despite the drawbacks of being costly and often involving onerous state-permitting requirements, GPS tracking technology may be the most appropriate method for reliable post-release monitoring to assess the success of applied rehabilitation programmes and help direct resources where they are most effective [50].

Given the likelihood that WRCs will become increasingly important in the future, the need for holistic, evidence-based guidelines for wildlife rehabilitation goes beyond recovery from an injury and post-release survival to also focus on the contribution of WRCs to the maintenance of reproducing populations and the survival of species through rehabilitated individuals.

## Figures and Tables

**Table 1:** Species-specific number and share of admission causes and rehabilitation efforts for wild birds admitted to NWBRC between 2005 and 2024. Only species with a minimum of 100 individuals (*n* = 14) are shown. Highlighted rows (grey) indicate higher-level systematic bird groups that include the respective species listed above. The total numbers of admissions summarised by bird group (highlighted rows) and admission cause (bottom row) also include individuals of other species not shown in the table, thus summarising the full data set of the study (*n* = 5102). The species-specific rehabilitation effort is presented as the cumulative duration of rehabilitation in days. Values are presented as the number of individuals per bird species/group, with the species-/group-related percentage in brackets.

Species	Build	Struct	Vehic	Trauma	Persec	Pet	Infect	Condit	Orph	Undet	TOTAL (%)	Rehabilitation Effort (%)
Common Buzzard	23 (10)	7 (3)	33 (15)	101 (45)	0 (0)	1 (0)	4 (2)	20 (9)	11 (5)	25 (11)	225 (4)	4316 (3)
Eurasian Sparrowhawk	33 (21)	4 (2)	18 (11)	50 (31)	0 (0)	0 (0)	4 (2)	2 (1)	13 (8)	36 (22)	160 (3)	2830 (2)
Northern Goshawk	22 (12)	16 (9)	14 (8)	66 (35)	2 (1)	0 (0)	8 (4)	10 (5)	21 (11)	27 (15)	186 (4)	3232 (2)
Accipitriformes	79 (13)	27 (4)	69 (11)	238 (39)	2 (0)	1 (0)	17 (3)	34 (6)	45 (7)	101 (16)	613 (12)	11,022 (7)
Carrion Crow	24 (4)	3 (1)	19 (3)	147 (26)	3 (1)	5 (1)	50 (9)	25 (4)	166 (29)	123 (22)	565 (11)	24,190 (15)
Eurasian Jay	7 (5)	1 (1)	5 (3)	30 (20)	2 (1)	3 (2)	12 (8)	6 (4)	44 (29)	43 (28)	153 (3)	4850 (3)
Eurasian Magpie	15 (8)	0 (0)	6 (3)	34 (19)	1 (1)	3 (2)	31 (17)	3 (2)	45 (25)	42 (23)	180 (4)	6144 (4)
Corvids	46 (5)	4 (0)	33 (4)	216 (24)	6 (1)	11 (1)	93 (10)	35 (4)	259 (28)	211 (23)	914 (18)	35,798 (23)
Common Kestrel	29 (8)	9 (3)	32 (9)	73 (20)	3 (1)	0 (0)	1 (0)	21 (6)	86 (24)	105 (29)	359 (7)	6542 (4)
Falconiformes	32 (9)	11 (3)	33 (9)	77 (21)	3 (1)	0 (0)	1 (0)	23 (6)	86 (23)	109 (29)	375 (7)	6734 (4)
Common Blackbird	23 (5)	1 (0)	13 (3)	72 (15)	0 (0)	32 (7)	43 (9)	11 (2)	144 (30)	136 (29)	475 (9)	12,228 (8)
Common Starling	6 (5)	1 (1)	2 (2)	15 (13)	1 (1)	2 (2)	7 (6)	8 (7)	27 (24)	45 (39)	114 (2)	2517 (2)
Great Tit	7 (5)	1 (1)	3 (2)	15 (10)	0 (0)	4 (3)	8 (5)	8 (5)	55 (35)	54 (35)	155 (3)	3401 (2)
Greenfinch	8 (6)	0 (0)	2 (1)	21 (15)	0 (0)	3 (2)	5 (4)	5 (4)	58 (41)	38 (27)	140 (3)	3877 (2)
House Sparrow	36 (7)	2 (0)	13 (2)	82 (15)	0 (0)	14 (3)	1 (0)	28 (5)	186 (35)	175 (33)	537 (11)	18,712 (12)
Passerines	144 (7)	8 (0)	46 (2)	282 (14)	5 (0)	69 (4)	71 (4)	88 (5)	608 (31)	627 (32)	1948 (38)	52,117 (33)
Long-Eared Owl	27 (26)	4 (4)	9 (9)	35 (33)	2 (2)	0 (0)	1 (1)	2 (2)	11 (10)	14 (13)	105 (2)	1379 (1)
Strigiformes	42 (22)	13 (7)	19 (10)	55 (28)	3 (2)	0 (0)	2 (1)	6 (3)	24 (12)	31 (16)	195 (4)	3005 (2)
Wood Pigeon	63 (6)	5 (0)	50 (5)	284 (27)	2 (0)	19 (2)	71 (7)	29 (3)	243 (23)	291 (28)	1057 (21)	50,065 (32)
Total	406 (8)	68 (1)	250 (5)	1152 (23)	21 (0)	100 (2)	255 (5)	215 (4)	1265 (25)	1370 (27)	5102 (100)	158,741 (100)

Note: Causes: “build”, building collision; “struct”, anthropogenic structure; “vehic”, vehicle collision; “trauma”, unknown trauma; “persec”, persecution; “pet”, pet attack; “infect”, infection; “condit”, poor overall condition; “orph”, orphaned; “undet”, undetermined. See Table S1 for full case definitions.

**Table 2:** Overview of the rehabilitation outcomes, durations, and total numbers for all admission causes of wild birds admitted to the NWBRC between 2005 and 2024. Values represent numbers for released, deceased/euthanised, and total individuals, with the percentage (%) given in brackets calculated for each admission cause. The total proportion is based on the full data set of admissions (*n* = 5102). * Rehabilitation duration is presented in days as median ± MAD (min–max) and based on released individuals (*n* = 3616). MAD, median absolute deviation.

Admission Cause	Released (%/Cause)	Deceased/Euthanised (%/Cause)	Rehabilitation Duration (Days *)	Total
Building collision	271 (67)	135 (33)	28 ± 19 (1–163)	406 (8)
Anthrop. structure	51 (75)	17 (25)	16 ± 18 (1–142)	68 (1)
Vehicle collision	165 (66)	85 (34)	38 ± 21 (3–174)	250 (5)
Unknown trauma	738 (64)	414 (36)	36 ± 22 (1–194)	1152 (23)
Persecution	11 (52)	10 (48)	39 ± 37 (9–99)	21 (0)
Pet attack	55 (55)	45 (45)	37 ± 19 (1–123)	100 (2)
Infection	188 (74)	67 (26)	47 ± 25 (1–186)	255 (5)
Poor overall condition	82 (38)	133 (62)	32 ± 28 (1–167)	215 (4)
Orphaned	1000 (79)	265 (21)	36 ± 22 (1–197)	1265 (25)
Undetermined	1055 (77)	315 (23)	37 ± 22 (1–194)	1370 (27)
Total admissions	3616 (71)	1486 (29)	36 ± 22 (1–197)	5102 (100)

**Table 3:** Summary of ringing metrics and post-release survival as a proxy for rehabilitation success (defined as at least two months of survival) of ringed rehabilitated birds (*n* = 1767, *n*_species_ = 12) admitted to the NWBRC between 2005 and 2024. Only species with ≥10 ringed individuals were considered, excluding 22 individuals from 12 species from the full data set of wild bird admissions.

		Ringing			Post-Release Survival		
Bird Group	Species	Ringed	Ringed + Recovered	Ring Recov. Rate (%)	Successful (%/Ringed)	Failed (%/Ringed)	Unknown (%/Ringed)
Accipitriformes	Common Buzzard	125	3	2.4	3 (2)	0 (0)	122 (97)
	Eurasian Sparrowhawk	94	1	1.1	0 (0)	0 (0)	94 (100)
	Northern Goshawk	73	5	6.8	3 (4)	2 (2)	68 (93)
Charadriiformes	European Herring Gull	20	0	0	0 (0)	0 (0)	20 (100)
Pelecaniformes	Grey Heron	15	4	26.7	4 (26)	0 (0)	11 (73)
Columbiformes	Wood Pigeon	641	3	0.5	0 (0)	1 (0)	640 (99)
Falconiformes	Common Kestrel	150	5	3.3	3 (2)	0 (0)	147 (98)
Corvids	Carrion Crow	351	1	0.3	1 (0)	0 (0)	350 (99)
	Eurasian Jay	92	1	1.1	1 (1)	0 (0)	91 (98)
	Eurasian Magpie	126	1	0.8	1 (0)	0 (0)	125 (99)
Strigiformes	Long-Eared Owl	54	0	0	0 (0)	0 (0)	54 (100)
	Tawny Owl	26	0	0	0 (0)	0 (0)	26 (100)
	Total	1767	24	1.3	16 (1)	3 (0)	1748 (99)

## Data Availability

The data and R code that support the findings of this study will be available on GitHub (https://github.com/marcengler/Engler_et_al_2025_Animals, accessed on 9 June 2025) and Zenodo (https://zenodo.org/records/15648030, accessed on 9 June 2025).

## Supplementary Materials

The following supporting information can be downloaded at: https://www.mdpi.com/article/10.3390/ani15121746/s1; Table S1: Admission type, cause and descriptions for wild birds admitted to the NABU wild bird station in Berlin (Germany) between 2005–2024 (n = 5102); Figure S1: Histogram showing the distribution of rehabilitation duration of wild bird admissions to the NABU wild bird station in Berlin (Germany) between 2005–2024; Table S2: Model parameter estimates for the GLM predicting rehabilitation duration based on the bird group and admission cause of wild bird admissions to the NABU wild bird station in Berlin (Germany) between 2005–2024; Table S3: Model parameter estimates for the GLM predicting release probabilities based on bird group and admission cause of wild bird admissions to the NABU wild bird station in Berlin (Germany) between 2005–2024.
