# Supplementary material for: Worth the Effort? Rehabilitation Causes and Outcomes and the Assessment of Post-Release Survival for Urban Wild Bird Admissions in a European Metropolis
Source: Animals (Basel). 2025 Jun 13;15(12):1746. doi: 10.3390/ani15121746 (PMC12189551; doi:10.3390/ani15121746)
Supplement: Supplementary file 1 [file animals-15-01746-s001.zip › animals-3652876-supplementary.pdf]

Supplementary material

Table S1: Admission type, cause and descriptions of wild birds admitted to the NABU wild bird station in Berlin (Germany) between 2005 and 2024 (n = 5102).

Admission cause	Description
Anthrop. structures	Admissions, including casualties through entanglement in any anthropogenic structures, i.e. fences, fishing lines, waste material, netting over ponds, cattle grids
Building collision	Admissions, including collisions with buildings or anthropogenic structures, birds falling down chimneys, trapped down wells or drains and trapped in sheds or greenhouses
Vehicle collision	Admissions, including collisions with vehicles such as cars or trains and stunned casualties found on roads
Unknown trauma	All casualties with physical injuries/wounds or those in shock for which the exact cause could not be determined
Persecution	Admissions including direct and indirect persecution, i.e. gunshot victims, poisoning and casualties through traps or snares
Pet attack	Admissions for which the finder observed casualties through attack by a domesticated animal (e.g., cat or dog)
Infection	Admissions diagnosed with clinical expression of parasitic or bacterial infection
Poor overall condition	Admissions with signs of dehydration and/or starvation and/or apathy
Orphaned	Admissions of nestlings or fledglings outside the nest
Undetermined	Casualties without physical injuries/wounds, not in shock and for which the exact cause could not be determined

Figure S1: **Histogram showing the distribution of rehabilitation duration of wild bird admissions to the NABU wild bird station in Berlin (Germany) between 2005 and 2024.** The data only includes individuals who went through the full rehabilitation process, i.e. were released (n = 3616). The red line represents the mean rehabilitation duration. Note that in the final data set used to model rehabilitation duration based on admission cause and bird group, admission records with a duration of > 200 days were excluded.

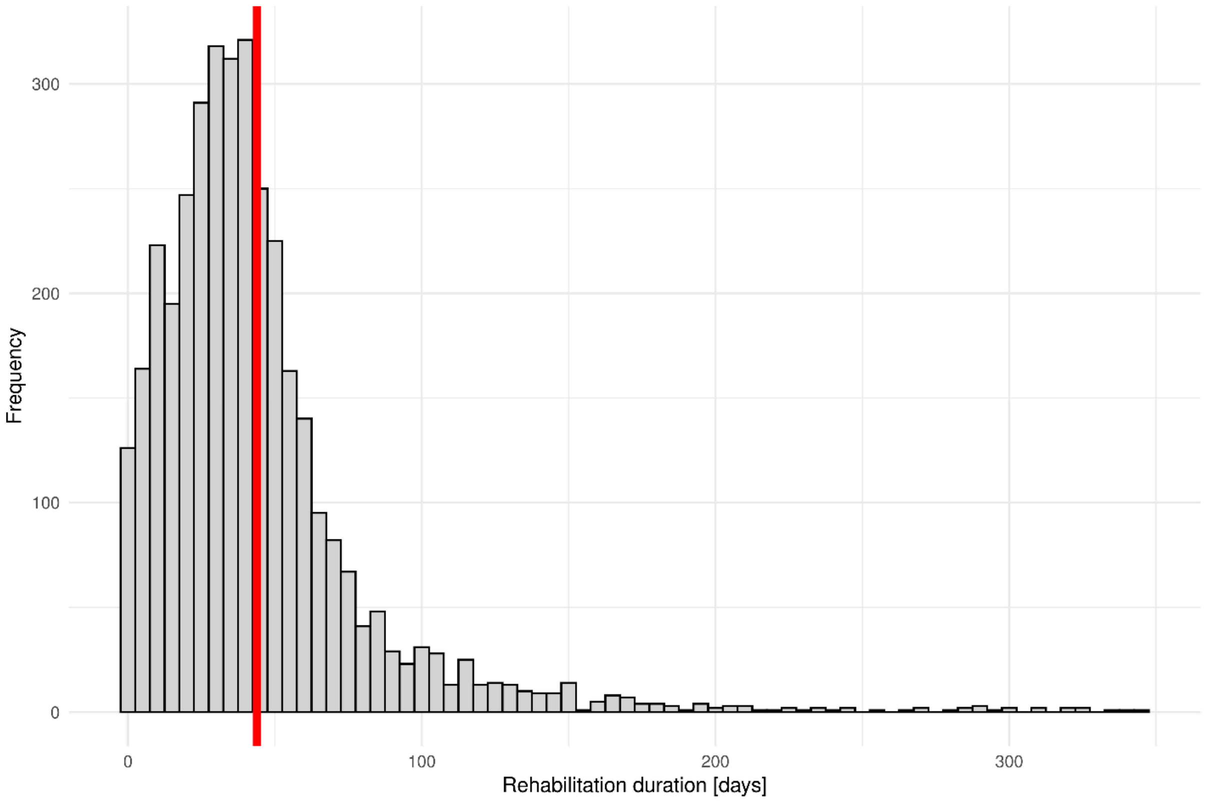

Table S2: Model parameter estimates for the GLM predicting rehabilitation duration based on the bird group and admission cause of wild bird admissions to the NABU wild bird station in Berlin (Germany) between 2005 and 2024. The model was fitted on a dataset including only individuals undergoing the full rehabilitation process (i.e. until successful release, n = 3576). The intercept corresponds to the baseline where 'undetermined is the reference level for "admission cause" and 'Passerines the reference level for "bird group". Standard Errors for parameter estimates are displayed as conditional estimates.

Variable	Estimate	Cond. SE
(Intercept)	3.658	0.03
admission_causeanthropogenic_structure	-0.504	0.335
admission_causebuilding_collision	-0.43	0.075
admission_causeinfection_parasites	-0.031	0.094
admission_causeorphaned	-0.023	0.043
admission_causepoor_overall_condition	0.053	0.137
admission_causeunknown_trauma	-0.016	0.057
admission_causevehicle_collision	0.159	0.127
groupRaptors	-0.896	0.057
groupCorvids	0.391	0.062
groupWood_pigeon	0.471	0.052
admission_causeanthropogenic_structure:groupRaptors	0.759	0.355
admission_causebuilding_collision:groupRaptors	0.99	0.109
admission_causeinfection_parasites:groupRaptors	0.405	0.208
admission_causeorphaned:groupRaptors	-0.251	0.089
admission_causepoor_overall_condition:groupRaptors	0.183	0.195
admission_causeunknown_trauma:groupRaptors	0.572	0.086
admission_causevehicle_collision:groupRaptors	0.534	0.154
admission_causeanthropogenic_structure:groupCorvids	0.065	0.575
admission_causebuilding_collision:groupCorvids	-0.103	0.174
admission_causeinfection_parasites:groupCorvids	0.017	0.134
admission_causeorphaned:groupCorvids	-0.142	0.082
admission_causepoor_overall_condition:groupCorvids	-0.205	0.233
admission_causeunknown_trauma:groupCorvids	-0.084	0.102
admission_causevehicle_collision:groupCorvids	-0.246	0.206
admission_causeanthropogenic_structure:groupWood_pigeon	0.803	0.467
admission_causebuilding_collision:groupWood_pigeon	0.256	0.132
admission_causeinfection_parasites:groupWood_pigeon	0.121	0.138
admission_causeorphaned:groupWood_pigeon	0.013	0.076
admission_causepoor_overall_condition:groupWood_pigeon	0.1	0.212
admission_causeunknown_trauma:groupWood_pigeon	-0.005	0.085
admission_causevehicle_collision:groupWood_pigeon	-0.189	0.175

Table S3: Model parameter estimates for the GLM predicting release probabilities based on bird group and admission cause of wild bird admissions to the NABU wild bird station in Berlin (Germany) between 2005 and 2024. The model was fitted on the full dataset of 5102 individuals. The intercept corresponds to the baseline where ‘undetermined is the reference level for "admission cause" and ‘Passerines the reference level for “bird group”. Standard Errors for parameter estimates are displayed as conditional estimates.

Variable	Estimate	Cond. SE
(Intercept)	1.201	0.095
admission_causeanthropogenic_structure	-1.201	0.713
admission_causebuilding_collision	-0.57	0.199
admission_causeinfection_parasites	-0.045	0.294
admission_causeorphaned	-0.049	0.134
admission_causepoor_overall_condition	-2.182	0.257
admission_causeunknown_trauma	-0.587	0.157
admission_causevehicle_collision	-0.667	0.32
groupRaptors	0.217	0.188
groupCorvids	-0.347	0.178
groupWood_pigeon	0.147	0.173
admission_causeanthropogenic_structure:groupRaptors	1.194	0.812
admission_causebuilding_collision:groupRaptors	0.26	0.318
admission_causeinfection_parasites:groupRaptors	-0.525	0.592
admission_causeorphaned:groupRaptors	0.379	0.309
admission_causepoor_overall_condition:groupRaptors	0.541	0.396
admission_causeunknown_trauma:groupRaptors	-0.109	0.251
admission_causevehicle_collision:groupRaptors	0.069	0.409
admission_causeanthropogenic_structure:groupCorvids	0.347	1.238
admission_causebuilding_collision:groupCorvids	-0.547	0.388
admission_causeinfection_parasites:groupCorvids	0.247	0.406
admission_causeorphaned:groupCorvids	0.676	0.257
admission_causepoor_overall_condition:groupCorvids	0.802	0.46
admission_causeunknown_trauma:groupCorvids	-0.23	0.256
admission_causevehicle_collision:groupCorvids	0.118	0.499
admission_causeanthropogenic_structure:groupWood_pigeon	1.239	1.334
admission_causebuilding_collision:groupWood_pigeon	-0.085	0.363
admission_causeinfection_parasites:groupWood_pigeon	-0.367	0.421
admission_causeorphaned:groupWood_pigeon	0.103	0.255
admission_causepoor_overall_condition:groupWood_pigeon	1.182	0.479
admission_causeunknown_trauma:groupWood_pigeon	0.04	0.249
admission_causevehicle_collision:groupWood_pigeon	-0.018	0.461
